# Empowering sex workers? Critical reflections on peer-led risk-reduction workshops in Soweto, South Africa

**DOI:** 10.1080/16549716.2018.1522149

**Published:** 2019-01-15

**Authors:** Susann Huschke

**Affiliations:** Graduate Entry Medical School, University of Limerick, Ireland, & School of History, Anthropology, Philosophy and Politics, Queen’s University Belfast, Belfast, UK

**Keywords:** Sex work, South Africa, empowerment, participation, peer education, risk reduction, conscientization

## Abstract

**Background**: Sex workers in South Africa face various forms of structural and interpersonal violence, including police violence, exclusion from health services, and stigmatization and marginalization within their communities. In an attempt to counteract the harmful health effects of criminalization and exclusion, risk-reduction workshops are a key component of HIV prevention programs globally. This paper offers a critical investigation of Creative Space workshops – a South African model of risk-reduction workshops for sex workers – taking place in Soweto, Johannesburg. Drawing on Paulo Freire’s work, the paper explores the potential of these workshops to contribute to the empowerment, health and well-being of sex workers.

**Objectives**: The aim of this paper is to investigate the social and psychological effects of peer-led risk-reduction workshops for sex workers in Soweto, South Africa, with a particular focus on the ways in which they might contribute to community empowerment.

**Methods**: This paper is based on in-depth interviews and focus group discussions with 32 sex workers conducted as part of a 20-month ethnographic study (December 2015 to July 2017). Data was analyzed combining inductive thematic analysis with a theoretical frame based on Freire’s theory of community empowerment.

**Results**: Peer-led risk-reduction workshops can serve as a ‘safe space’ for sex workers and distribute empowering forms of knowledge, particularly regarding health issues and rights. However, divisions between different groups of sex workers and between sex workers and non-sex workers counteract the potential benefits of the workshops.

**Conclusions**: Peer-led sex worker programs are likely to be more empowering when they are committed to raising critical consciousness and creating solidarity, and embedded in community action, focusing on common issues such as institutionalized racism, livelihood insecurity, and lack of access to safe and secure housing. Such actions would have positive outcomes on health and well-being.

## Background

Sex work is criminalized in South Africa. As a result, sex workers face various forms of structural and interpersonal violence that negatively affect their health and well-being. This includes police violence, exclusion from health services, stigmatization and marginalization within their communities, violence from clients, and – as a result – a higher likelihood of acquiring HIV than the general population [1–3]. In an attempt to counteract the harmful health effects of criminalization and exclusion, risk-reduction workshops for sex workers are a key component of HIV prevention programs globally [4–6]. In South Africa, risk-reduction workshops – locally known as Creative Space workshops (CSWs) – were rolled out across the country in 2010 as an initiative of the Sex Workers Education and Advocacy Task Force (SWEAT), a non-governmental organization working to support the health and rights of individuals working in the sex industry. The aims of CSWs include education of sex workers, particularly regarding sexually transmitted infections (STIs); promotion of HIV counselling and testing (HCT); networking and community building; psychosocial support; and sensitization of key stakeholders who can be invited to the workshops, such as local police [7]. CSW sessions are generally led and facilitated by peer educators; that is, by current or former sex workers who received basic training in group facilitation and STI prevention from SWEAT. As such, they are part of the global sex worker movement centered on the idea of ‘nothing about us without us’.

**Figure 1.**
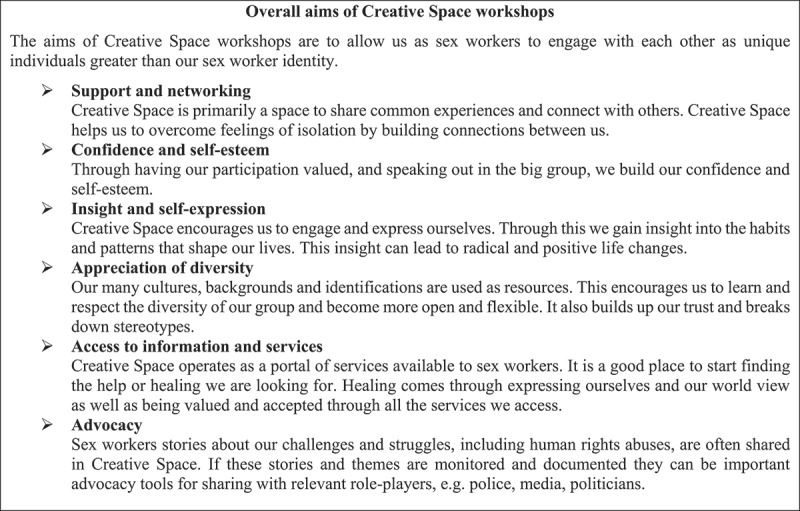
Overall aims of Creative Space workshops. [22, p. 5].

Globally, similar peer-led workshops have been used as a risk-reduction and STI prevention intervention with sex workers [5,8–12] and other ‘high-risk’ groups such as transgender people [13], injecting drug-users [14], and young people [15,16]. Generally, evaluations of these peer-led interventions focus on their effects regarding STI knowledge, STI and HIV rates, condom use, and use of HCT, with mixed results indicating that while peer education programs can be effective medical interventions, they are not a bullet-proof method [8,9,17–21]. The psychological and social effects of peer-led workshops have received much less attention in the existing empirical studies [17,20]. However, they constitute key aims of sex worker-led risk-reduction workshops, according to the SWEAT CSW manual, which specifically mentions, for example, ‘overcoming feelings of isolation’, ‘connecting with others’, and ‘building confidence and self-esteem’ (see Figure 1).

The aim of this paper is to address this research gap by investigating the social and psychological effects of Creative Space workshops, with a particular focus on the ways in which CSWs might contribute to community empowerment.

## Conceptual frame: empowerment

The driving principle behind peer-led harm and risk-reduction interventions with sex workers locally [23] and globally is *community empowerment* [17,24,25]. This liberatory educational approach developed by the Brazilian educator Paulo Freire informs participatory interventions across the globe [26,27]. One of the key components of empowerment, according to Freire, is *conscientization*, the process of raising individual and collective critical consciousness regarding the social, economic, and political structures that shape people’s lives [28]. Freire posits that action and reflection need to be linked: a collective understanding of the social circumstances people find themselves in needs to lead to collective action *upon* these circumstances for people to be empowered [29]. Hope and solidarity are key experiences in this process of empowerment. Becoming part of a collective, developing a critical understanding of the concrete local social context that shapes the experiences of individuals, and practicing solidarity all nurture hope, which in turn is the prerequisite for any struggle [30]. In this paper, I draw on Freire’s conceptualization of empowerment to analyze sex workers’ views on the potential and shortcomings of Creative Space workshops. The focus on empowerment is grounded both in the empirical data – empowerment constituting one of the declared aims of interventions such as CSWs – and my theoretical and methodological approach which was informed by Freire’s work.

## Local context of the study

In this paper, I focus specifically on the experiences of sex workers who participate regularly in the CSWs run by the Soweto Sex Worker Programme (SSWP) located at the Perinatal HIV Research Unit (PHRU) in Baragwanath Hospital, Soweto. The project receives external funding with the overall aim to reduce HIV rates among sex workers in Soweto. Soweto is one of South Africa’s largest townships, with a predominantly Black population. At the SSWP Creative Space, participants are given lunch and R40 (approx. US$3) as a reimbursement for their time. The workshops are offered on a monthly basis and are run by the peer educators – themselves current or former sex workers – who are employed at the site. They report to the program coordinator (who is not a sex worker), in charge of the overall content of the interventions delivered by SSWP. The workshop facilitators usually ask the participants if there are any topics that they would like to discuss, while at the same time being prepared to give inputs on certain topics, most commonly health-related issues such as the benefits of pre-exposure prophylaxis (PrEP) or the importance of condom use. Occasionally, the facilitators invite external experts to participate in the Creative Space sessions; for example, representatives of the local Women’s Legal Centre who can answer questions regarding reporting violence, opening a court case, labor rights, and domestic law.

## Methods

### Study design

This paper is based on an analysis of a subset of data gathered in a 20-month ethnographic study on the health and well-being of sex workers in Soweto between December 2015 and July 2017. The larger study incorporated two phases. During the first phase of the study, I interviewed female and transgender sex workers on issues related to health and well-being, including experiences of violence and stigmatization and coping strategies, and participated in SSWP outreach activities and Creative Space sessions (n = 20). In the second phase of the study, I focused on facilitating Know My Story, a participatory arts-based project with 14 sex workers [2], 8 of whom had been interviewed in the previous phase. To explore the differences and similarities between local and internal migrant sex workers in more depth, four focus group discussions were conducted in isiZulu by the research assistant (n = 12).

#### Data subset

This paper specifically focuses on sex workers’ experiences with Creative Space Workshops. The question – in which ways CSWs may contribute to social and psychological well-being and to community empowerment – arose organically over the course of the larger study. Many of the sex workers who took part in this study brought up CSWs in our conversations, highlighting the importance of these workshops in regard to their sense of self-worth and belonging to a larger sex worker community, but also addressing frictions and shortcomings. This led me to re-analyze all of the interview transcripts specifically focusing on sex workers’ views on and experiences with Creative Space, using NVivo software. I furthermore conducted follow-up, topic-centered interviews with previous interviewees who were also peer educators facilitating Creative Space sessions (n = 4), discussing the benefits and problems of Creative Spaces as interventions.

The findings presented in this paper are grounded in my analysis of this subset of all data, namely the in-depth interviews with 20 sex workers conducted at the start of my research, the 4 additional follow-up interviews with workshop facilitators, and the 4 focus group discussions with a total of 12 local and internal migrants sex workers (who had not been interviewed previously).

#### Participant demographics

All of the 32 research participants whose experiences were analyzed for this paper were Black South Africans, including in total 17 internal migrants from the Eastern Cape, Kwa-Zulu Natal, and Limpopo provinces. The age of the participants ranged from 22 to 50 years. The participants had been selling sex for between one year and three decades. One of the interviewees identified as transgender, all others identified as women. All names are pseudonyms to ensure anonymity.

#### Sampling and analysis

Convenience sampling was employed to recruit interviewees. I met most of the interviewees either in their role as SSWP peer educators or as Creative Space participants. Other interviewees were contacted via snow-balling, i.e. through other research participants. Interviews were transcribed verbatim. The focus group interviews that were conducted in isiZulu were translated into English for analysis by the research assistant. The themes explored in this paper – competition, jealousy, solidarity, and creative space as a healing environment – gradually emerged over the course of the research as ‘grounded findings’ [31], based on open in-vivo coding of the interview data and field notes parallel to field work, and on triangulation of all data. The follow-up interviews with peer educators served to discuss the themes that had arisen from the previous phases of the research, and helped reach data saturation in regard to the specific questions addressed in this paper.

## Results

In the following, I present the main themes that arose from the interviews and focus group discussions with sex workers on their experiences with Creative Space workshops. I describe the knowledge transfer between sex workers and the impact of CSWs on sex workers’ emotional and psychological well-being as two important positive aspects. At the same time, participants highlighted shortcomings, including the disruptive effect of jealousy and competition between sex workers, the fear that participating in CSW might lead to involuntary disclosure outside the workshop space, and the sense that there is little room within CSWs to talk about the desire to *exit* the sex industry.

### ‘Now I know where to go’: knowledge transfer

One of the crucial effects of Creative Space workshops described by participants and facilitators alike is the transmission of knowledge, particularly regarding sexual health, sex worker rights, and existing support structures. Zenande, a sex worker from the Eastern Cape, pointed out:

[At Creative Space] I heard of words such as human rights and decriminalization. I learnt […] of various organizations and what they can offer a sex worker like me. I was taught about ARVs [antiretrovirals] and the importance of testing for me, and the dangers of not using protection even in simple blowjobs. (Interview 6 July 2017)

Addressing the violence sex workers in South Africa experience, Chantel, a Creative Space facilitator, explained:

Going to Creative Space, that’s what they taught us: you can also open a case against the police; you can also open a case against clients. Because we thought if a client came and raped you, you just let it go […]. So going to Creative Space changed me a lot. Now I know, if someone messes with me, I know where to go, I know what to do. (Interview 6 July 2017)

However, interviewees also felt that there are limitations to the knowledge that can be shared in a sex worker-only space, and suggested that medical and legal experts should be invited more frequently to the workshops. Alternatively, sex workers suggested that they should receive more training to be able to respond, for example, to specific medical and legal questions – a point that relates to the critique discussed further below regarding the need for skills development as a way of creating alternatives to sex work.

### ‘When I come here, I feel better’: creative space as a healing space

One of the most important positive aspects of Creative Space workshops is the effect it can have on the emotional and psychological well-being of the participants. Interviewees explained how participating in Creative Space had increased their ability to cope with past and present experiences of (sexual) violence, exclusion, and stigmatization. In a group discussion with internal migrant sex workers, Ntombi, an internal migrant from Kwa-Zulu Natal, mentioned:

That’s why I like coming to Creative Space, because when I’m stressed I [used to] like to be alone. I grew up suicidal. I’ve attempted suicide more than ten times. But I don’t want to do it anymore. I was even telling my boyfriend that when I come here, I feel better. (Interview 16 June 2017)

Pretty, a 28-year-old woman from Soweto, was raped when she was 11 years old. At Creative Space, she talked about how this experience of violence changed her forever, and about the uncontrollable anger that she feels as a result of it. When I interviewed her a few weeks later, she explained the role of Creative Space in her healing:

It is helping me. It helped because I want to move on with my life. I want to take the past away […]. Every time when I am busy talking, talking, I am healing inside […]. I am busy healing myself in fact. (Interview 28 June 2016)

Lihle, a 34-year-old woman from Soweto, stressed the importance of talking to people who can relate to your experiences and do not judge you for being a sex worker:

‘Not being judged – it’s out of this world, because you even say things you thought you could never say to anyone. You become free […]. You forget some of the hectic stuff. You become light’ (Interview 6 July 2017).

These quotes clearly indicate the transformative, healing aspect of Creative Space sessions for participants on an individual level. However, the interviews also brought to light that frictions and conflicts are a recurrent feature of Creative Space workshops, as I show in the next section.

### ‘In this work, there are no friends’: competition and jealousy

One of the key issues raised by both migrant and local sex workers is the competition and jealousy between different groups of sex workers. These interpersonal frictions can reduce the positive effects of Creative Space workshops on sex workers’ well-being, or even lead to new experiences of physical violence, as Rethabile from Durban highlighted:

‘When people from here know that you are not from around here, they take advantage of you. Others get angry and end up hitting you because this is their territory’ (Interview 16 June 2017).

Migrant sex workers often face discrimination from local sex workers, who may, for example, encourage clients to pay migrant sex workers less and ridicule them in front of clients. However, the interviews revealed that rivalry and (sometimes violent) competition between sex workers is common, even between sex workers from within Soweto. For example, if a sex worker who usually works in Diepkloof in the east of Soweto decides to look for clients in Naledi in central Soweto, s/he is likely to not be welcomed by established sex workers:

There’s no such thing as being family [of sex workers] because we fight amongst ourselves. If I’m new, your clients will buy me because I’m new, and then you get angry, there will be a fight. […] In this work there are no friends. (Focus group interview 23 June 2017)

These divisions can play into Creative Space sessions and jeopardize the empowering atmosphere. The peer educators interviewed were very aware of these issues, as Chantel explained:

That’s what we teach: for them to work together, to make friends, so that, let’s say, you are moving from Diepkloof, you want to go to Jabulani Hostel, so that you can go there and feel free to work. [We try] to make them work together so that we can stop the stigma and the abuse. (Interview 6 July 2017)

To an extent, interviewees felt that the peer educators are successful in their attempt to ‘make them work together’. For example, Zenande highlighted how Creative Space encouraged her to be kinder and more supportive of fellow sex workers:

When one is doing sex work, we learn to be greedy, grumpy, self-centered and jealous, and at the same time very ashamed of the person one has become. Creative Space takes all the negative energy away; it’s a space that says: you are doing sex work because you choose to. You are not alone. We are all here as sex workers to share your fears, highlights, and empowerment. (Interview 6 July 2017)

However, it is sometimes difficult to ensure that the space is reserved for sex workers, as I discuss in the next section.

### ‘People will judge me’: when the safe space is not safe

Stigmatization is a constant threat for sex workers. Sharing experiences and outing themselves as sex workers to dozens of people in a Creative Space session puts participants in a vulnerable position, and some interviewees expressed concerns regarding confidentiality. Amahle, a 24-year-old woman from Soweto, explained that while she feels sharing stories in Creative Space helps sex workers cope with their experiences, she would not feel comfortable sharing her own story. She said:

‘[Creative Space helps] because you confront whatever hurts you. But I don’t think I’ll tell them my story. It’s not easy and I think people will judge me and will go outside and talk’ (Focus group interview 23 June 2017).

Further explorations of this theme in the interviews revealed that the main concern is that non-sex workers might be present in the room. Sex workers worried that non-sex workers might start gossiping about the personal stories shared in the space and disclose their source of income to their communities. Zenande explained:

My fear with Creative Space, as much as I like it, is that, sex workers have friends that are not sex workers and take them along. [And] they would see me, and they would say [to others]: ‘she’s a sex worker.’ […] Simply because that person is not a sex worker herself, and has not taken time to listen and to understand. (Interview 20 April 2016)

Similarly, peer educator Lihle discussed this issue, which seemed to have become more prevalent in the year between the interview with Zenande and the interview with Lihle:

‘[Creative Space] has become a platform for people. People who are hungry come there, people who are not even aware of what is happening there. People take their friends along: “let’s go, we’re gonna get 40 Rands.” ’ (Interview 6 July 2017).

The peer educators recently decided to respond to this ‘invasion’ of their space by screening participants, asking them detailed questions about where and how they (supposedly) work as sex workers, and turning away those who could not respond to the questions appropriately and were not known to any of the sex workers present.

### ‘There is no upskilling’: exiting the sex industry

In addition to the tensions discussed above that jeopardize the attempt to create a safe, empowering space that contributes to the health and well-being of the participants, some sex workers were disappointed that the discourse presented in Creative Space sessions focuses mainly on reframing sex work as acceptable work and on claiming a positive sex worker identity. Lihle, for example, expressed that she would like to be able to say that she does not like doing sex work, but that she feels that there is no space for that in the workshops:

‘In this creative space there is this mind set, like they don’t want us to say “I don’t like it [sex work].” It’s like it’s all about helping others to know that sex working is good’ (Interview 23 July 2016).

Similarly, Zenande explained that she would like to see concrete discussions about exiting the sex industry, and workshops that contribute to the upskilling or re-skilling of sex workers who would like to quit working or who are too old to be able to make a living by selling sex:

Exiting seems distant and undoable. There won’t be anything else when one becomes stale, wants out or has grown grey and old. There’s no references, let alone a CV compilation for the sex work done. The government refused to decriminalize sex work yet the government isn’t prepared to say this is what we are offering you to better your lives. [There is no] upskilling for me and my fellow colleagues. (Interview 6 July 2017)

## Discussion

In this paper, I set out to investigate the impact CSWs may have on the social and psychological well-being of sex workers, and how they may contribute to community empowerment. As discussed below, CSWs create a healing space for sex workers and facilitate knowledge exchange in relation to, for example, health and legal issues. At the same time, sex workers also offered a critique of CSWs, highlighting that the potential of these workshops to empower individuals – as well as the sex worker community as a whole – is not fully realized.

### A healing space

The experiences shared by sex workers stress the healing effect of Creative Space workshops, echoing the findings of previous research on participatory projects and peer-led interventions with sex workers more generally [4,17,32] and Creative Spaces more specifically [7,32]. Sex workers acknowledged the role of the group sessions in healing personal trauma and providing psychosocial support, and explained how they started feeling a sense of belonging and acceptance through participating in the workshops. Similar to other peer-led interventions such as the Sonagachi Project in Kolkata, India, the Creative Space workshops in Soweto inspire hope – hope that the conditions under which sex is sold can be changed:

‘In contrast to the expressions of fatalism […] in which discrimination against sex work appeared inevitable, by invoking the notion of rights, such discrimination is seen as illegitimate and the alternative – where their rights are duly respected – is conceivable’ [21, p. 466].

This process of reframing what it means to be a sex worker – from feeling ashamed to asserting rights – arguably reduces internalized stigma and creates a sense of self-worth. Many sex workers feel that these healing processes increase their emotional well-being. These interactions furthermore contribute to empowerment in the Freirean sense, as sex workers critically reflect on the structural forms of exclusion that drive the violence they experience.

### Knowledge and skills development

Knowledge acquisition constitutes another important contribution of Creative Space workshops. Sex workers stated that through Creative Space, they had been exposed not only to health-related knowledge, but also to information regarding their rights vis-à-vis the police, clients, and medical professionals. This translates into greater agency and empowers sex workers to challenge the injustices they face. As Chantel concisely put it: ‘[Now] I know where to go, I know what to do.’ However, some sex workers also felt that the factual knowledge shared in the Creative Space sessions, particularly in relation to medical and legal issues, is not sufficient, and suggested that experts, such as nurses and lawyers, should be invited on a more regular basis.

Furthermore, some sex workers felt that there was a quietly enforced emphasis on reframing sex work as a worthwhile way of making a living – in a way this constitutes the ideological counterpoint to widespread interventions that cast sex workers as helpless victims in need of rescue [33]. While the reframing of sex work in CSWs on one hand contributes to the destigmatization of sex work and is often experienced as empowering and healing, it can also serve as a way of silencing those who would like to leave the sex industry and have a need for information on how to facilitate this process. A Freirean approach to community education would focus on ‘generative themes’; that is, the ‘ideas, values, concepts and hopes, as well as the obstacles’ that matter to the people in question at this specific historical moment [34, p. 73]. It would be crucial for program coordinators to listen and respond to the actual – and clearly diverse – needs of sex workers, including the hope to leave the sex industry and earn money in other ways. This issue furthermore points to an important gap both in research and in harm-reduction interventions, which tend to focus on people who identify as sex workers, neglecting those who do sell sex but do not consider themselves to be sex workers and are reluctant to frame their income-generating strategies as a ‘profession’ [35].

### Structural factors limiting community empowerment

It is important to consider the context of the SSWP program (as described in the section on the local context above) as a potentially limiting factor for the empowerment of sex workers. Even though the peer educators run the Creative Space sessions autonomously and determine the content and form of the workshops, the program also involves non-community administrators and managers who hold significant power in regard to decision-making. This institutional set-up is likely to affect the potential for sex worker empowerment, defined in Freirean terms as the community’s capacity to define the issues that affect them and collectively develop responses to address these issues.

It is questionable to what extent peer-led Creative Space workshops embedded in a donor-funded public health project can actually lead – or are meant to lead – to the kind of empowerment and radical transformation of power relationships and social structures that the Freirean approach envisions [26]. Moore et al. [36] discuss the Sonagachi Project in West Bengal, India, as a global role model for community empowerment of sex workers. In their systematic review of sexual and reproductive health (SRH) interventions that target female sex workers in Africa, they found that most sex work projects – and I would argue that this applies to SSWP as well – are based on a biomedical model of disease control with the aim of changing the behaviour of individuals to reduce HIV and STIs. They are not rooted in a community development approach that aims to change the underlying social and political structures in order to improve sex workers’ health, including a collective struggle for decriminalization [36, p. 11].

### Mutual trust and solidarity

Sex workers also critically discussed the distrust, competition, and lack of solidarity that sometimes emerges in the workshops, mirroring the complex, often strained relationships between sex workers outside of the Creative Space context [17,20,32,37]. Interviewees felt that the hostility between different groups of sex workers – for example, between local and migrant sex workers – and between sex workers and non-sex workers who participate in the workshops to receive the reimbursement and the free lunch counteracts the healing effect of this ‘safe space’ and negatively affects the emotional well-being of the participants. This aspect has not received much attention yet in the literature evaluating peer-led risk-reduction programs. For example, in a recent evaluation of Creative Spaces across South Africa the authors concluded:

‘The only real challenge that was expressed was the high demand for the workshops and the limited resources available, which makes it difficult for sites to meet the demand’ [7,p.61].

The solutions developed by the Soweto sex workers to address these conflicts included interventions by the facilitators to exclude those who were not recognized as sex workers by the group. However, these tensions also reflect larger social, economic, and political issues that would be worth exploring as I explain in my conclusion.

### Limitations of the study

The findings presented here were developed inductively over the course of a 20-month research project. The focus of the larger study was not specifically on CSWs, and I did not conduct a systematic evaluation of CSWs for sex workers in South Africa. My findings could, however, constitute a starting point for more in-depth explorations of CSWs. They indicate that some of the existing frictions and failures of these interventions were missed in the recently conducted evaluation of CSWs [7]. However, it is also important to consider that my analysis is limited to one location, Soweto. To understand the potential and shortcomings of CSWs across the country, more research in other locations would be needed.

## Conclusion: taking action, creating solidarity

In this paper, I have drawn on interviews with sex workers in Soweto to discuss the empowering potential of peer-led risk-reduction workshops, referred to in South Africa as Creative Space workshops. Univocally, sex workers acknowledged the healing effect of peer-led group sessions, in which participants share experiences of their personal struggles and traumas. Creative Spaces contribute to sex workers’ sense of self-worth through a supportive, non-judgmental, rights-based discourse that allows for a reframing of sex work as work and a recasting of sex workers as citizens imbued with rights. They also expose sex workers to health knowledge, expand their understanding of their legal rights, and provide information on available support services. In this way, Creative Space workshops contribute to a greater sense of emotional well-being, and they are experienced as empowering: they increase sex workers’ sense of agency in the face of omnipresent structural and interpersonal violence, and create hope that things do not need to stay as they are.

At the same time, interviewees made clear that Creative Space is not a perfect social bubble in which the conflicts that sex workers experience in their daily lives are suspended. One of the key issues highlighted by the research participants was the tensions between different groups of sex workers and between sex workers and non-sex workers who sometimes participate in the workshops because there is free food and a small cash reimbursement. Other researchers have argued that peer education programs are more likely to be empowering if they connect to or are embedded in community-based initiatives that ‘address macrosocial problems – such as migrant labor, poverty, and sexual inequality’ [17,pp. 1985–1986, 38].

In regard to the conflict between sex workers and non-sex workers at the Soweto Creative Space, one could propose that while it seems indeed important to create spaces for sex workers only, there is also potential for wider community action, focusing on common issues such as poverty and institutionalized racism and creating solidarity networks. Equally, this could apply to different groups of sex workers, such as migrants and non-migrants: raising critical consciousness regarding the underlying causes of migration, such as the devastating effects of neoliberal capitalism on working-class communities particularly in rural areas, could lead to greater solidarity and mutual support within the sex worker community. Again drawing on the example of Sonagachi [4,20,36], I would argue that in order to truly contribute to community empowerment – which in turn has been shown to impact on the health and well-being of sex workers, including reduced HIV rates – programs such as Creative Spaces need to aim to hand over managerial power to the sex worker community in the long term, and need to be embedded in larger social struggles for equality.

## Paper context

While the effects of peer education programs on STI rates and health knowledge of sex workers have been documented in a number of studies, the relevance of these programs for the *empowerment* of sex workers has received less attention. In this paper, I show that risk-reduction workshops increase sex workers’ sense of agency in the face of omnipresent structural and interpersonal violence. However, the workshops also need to address the tensions between different groups of sex workers and non-sex workers and connect to wider struggles for social justice in order to increase the positive effects on the health and well-being of the participants.
